# Supplementary material for: Content-rich biological network constructed by mining PubMed abstracts
Source: BMC Bioinformatics. 2004 Oct 8;5:147. doi: 10.1186/1471-2105-5-147 (PMC528731; doi:10.1186/1471-2105-5-147)
Supplement: Additional File 5 — The original Chilibot query results of the term "long-term potentiation (LTP)" and 22 other terms, limiting the latest references analyzed to the years 1990, 1995, 2000, and 2004. [file 1471-2105-5-147-S5.bz2 › chilibotAdditionalFile5/ltp2004/html/ERK_PKA.html]

 


 **ERK** and **PKA** 
  
Found 189 abstracts in PubMed,  **30 abstracts were retrieved and analyzed**.  


---

 Search Google  |
 PDF files only 
|  EDU domain only 

---

**Interactive relationship** (e.g. stimulation, inhibition, etc)

- Forskolin blocked  **ERK**  phosphorylation with an EC50 of < ; 3 microm, and the protein kinase A  **PKA**  inhibitor H 89 enhanced  **ERK**  phosphorylation.  Ref: 14517212 J Biol Chem, 2003
- **ERK**  inhibition of long isoforms is regulated by a unique feedback control whereby elevated cAMP levels cause  **PKA**  protein kinase A to phosphorylate UCR1 and ablate the inhibitory action of  **ERK** .  Ref: 14641023 Biochem Soc Trans, 2003
- **ERK**  phosphorylation was strongly reduced by cortical infusion with the cAMP  **PKA**  inhibitor Rp 8 Cl cAMPS, thus establishing a link between  **PKA**  and  **ERK**  activation.  Ref: 12904462 J Neurosci, 2003
- p  **ERK**  level an initial decline in p  **ERK**  level followed by a sustained induction at 12 24 h, both of which were blocked by  **PKA**  inhibitor.  Ref: 14675145 J Neurochem, 2004
- Inhibition of  **PKA**  impaired context and tone dependent fear conditioning and significantly reduced the phosphorylation of Elk 1, p90Rsk 1,  **Erk**  1 2, and CREB.  Ref: 14739006 Behav Brain Res, 2004
- visual activation of  **ERK**  involves the cAMP  **PKA**  system.  Ref: 12904462 J Neurosci, 2003
- Finally, because CRE mediated gene expression was totally dependent on  **ERK**  activation, we suggest that  **PKA**  action on CRE mediated gene expression is mediated by  **ERK** .  Ref: 12904462 J Neurosci, 2003
- Moreover, protein kinase A  **PKA**  activity is required for depolarization induced Rap1 activation and full  **Erk**  stimulation, but is not involved in that of Ras.  Ref: 14597235 Brain Res Mol Brain Res, 2003
- These results suggest that PACAP mediated stimulation of  **ERK**  activity and proliferation of astrocytes may involve a cyclic AMP dependent, but  **PKA**  independent, pathway.  Ref: 14592419 Biochem Biophys Res Commun, 2003
- Phosphorylation by  **PKA**  prevents maximal coupling of the CRH R1alpha to Gq protein, and thereby reduces activation of  **ERK**  1 2.  Ref: 14657255 Mol EndocrinolMol Endocrinol, 2003
- This opposite regulation was specifically exerted by haloperidol and clozapine on  **ERK** , CREB, and Elk 1 phosphorylation, as both anti psychotic drugs increased the phosphorylation of the dopamine and cyclic AMP regulated phosphoprotein of 32 kDa DARPP 32 at the cyclic AMP dependent protein kinase  **PKA**  site.  Ref: 12871586 J Neurochem, 2003
- Interestingly,  **Erk**  1 2 phosphorylation after conditioning of nonstressed and stressed mice depended on  **PKA**  and Mek 1 2, respectively.  Ref: 14673008 J Neurosci, 2003

**Parallel relationship** (e.g. studied together, co-existance, homology, etc.)

- Prostaglandin E 2 PGE 2 and epinephrine act directly on nociceptors to produce mechanical hyperalgesia through protein kinase A  **PKA**  alone or through a combination of  **PKA** , protein kinase C epsilon PKCepsilon, and extracellular signal regulated kinase  **ERK** , respectively.  Ref: 12925276 Neuron, 2003
- KCl depolarization and forskolin synergistically activate the  **ERK**  signaling pathway, but whereas KCl mediated  **ERK**  activation depends on both  **PKA**  and MEK1, forskolin activates  **ERK**  in a  **PKA**  independent manner.  Ref: 12865141 Neurosci Lett, 2003
- Our results demonstrate that OxPAPC treatment activated in a time dependent fashion protein kinase C PKC, protein kinase A  **PKA** , Raf MEK  **Erk**  MAP kinase cascade, JNK MAP kinase and transient protein tyrosine phosphorylation in human pulmonary artery endothelial cells HPAEC.  Ref: 14709399 Microvasc Res, 2004
- We investigated the involvement of hippocampal protein kinase A  **PKA** , protein kinase C PKC, calcium calmodulin dependent protein kinases CaMK II, and mitogen activated extracellular signal regulated kinases 1 and 2 Mek 1 2 in the phosphorylation of their downstream targets extracellular signal regulated kinases 1 and 2  **Erk**  1 2, cAMP response element binding protein CREB, Elk 1, and p90 ribosomal S6 kinase 1 p90Rsk 1.  Ref: 14739006 Behav Brain Res, 2004
- The effects of KCl and forskolin were mediated via the protein kinase A  **PKA**  and the extracellular signal regulated kinase  **ERK**  signaling pathways, since addition of the  **ERK**  kinase MEK1 inhibitor PD98059 and the  **PKA**  inhibitor H89 inhibits neurite outgrowth.  Ref: 12865141 Neurosci Lett, 2003
- In some cases, like the  **ERK**  signaling pathway,  **PKA**  affects signal transduction by regulating the balance between specific phosphatases and kinases.  Ref: 14738140 Leuk Lymphoma, 2003
- These data implicate the inhibition of  **PKA**  as the predominant pathway for  **ERK**  activation by CB1 receptors in N1E 115 cells.  Ref: 14517212 J Biol Chem, 2003
- When PGE 2 hyperalgesia was shifted to dependence on PKCepsilon and  **ERK**  as well as  **PKA** , as when the tissue is primed by prior treatment with carrageenan, it too became dependent on an intact cytoskeleton.  Ref: 12925276 Neuron, 2003
- The circadian output pathway modulating CNGC channels is comprised in part of cAMP >  **PKA**  > Ras > B Raf > MEK >  **Erk**  > > CNGCs.  Ref: 14960600 J Neurosci, 2004
- To analyze the downstream consequences of  **ERK**  and  **PKA**  signaling, we studied the action of visual stimulation on transcription of genes controlled by CREB in transgenic mice carrying the LacZ reporter gene under the control of the CRE cAMP response element promoter.  Ref: 12904462 J Neurosci, 2003
- Recently, it has been shown that  **ERK**  extracellular signal regulated kinase 1 2, protein kinase A  **PKA** , and CREB cAMP response element binding protein are crucial factors for experience dependent development of the visual cortex, but very little is known about the role of visual experience in their activation.  Ref: 12904462 J Neurosci, 2003
- On the basis of these approaches, the cyclic AMP protein kinase  **PKA** , protein kinase C PKC and extracellularly regulated protein kinases 1 and 2  **Erk**  1 2 have been identified as the core signaling pathways in memory consolidation.  Ref: 14673206 NeurosignalsNeurosignals, 2003
- The results demonstrate that  **PKA**  and  **ERK**  play a key role in KCl and forskolin induced neuronal differentiation by integration of signals from both pathways.  Ref: 12865141 Neurosci Lett, 2003
- Standard protocols used to investigate the recognition of RGS proteins by 3 5 cyclic adenosine monophosphate cAMP dependent protein kinase  **PKA** , extracellular signal regulated kinase  **ERK** , and protein kinase C PKC are described, followed by strategies used to establish the specific amino acids modified by these kinases.  Ref: 14501052 Methods Mol Biol, 2004
- We also investigated the role of hippocampal corticotropin releasing factor receptor 2 CRF2 in stress enhanced learning and molecular signaling mediated by  **PKA** , Mek 1 2, and their downstream targets extracellularly regulated kinases 1 and 2  **Erk**  1 2 and p90 ribosomal s kinase 1 p90Rsk 1.  Ref: 14673008 J Neurosci, 2003
- Patterned vision causes CRE mediated gene expression in the visual cortex through  **PKA**  and  **ERK** .  Ref: 12904462 J Neurosci, 2003
- **Erk**  1 2, Elk 1, and CREB were identified as common targets of  **PKA** , PKC, and CaMK II during memory consolidation the time window and sequence of their phosphorylation was specific for the individual kinase.  Ref: 14739006 Behav Brain Res, 2004
- Here we show that cAMP, acting through protein kinase A  **PKA** , Ras, and  **Erk** , is part of the circadian output pathway controlling CNGCs.  Ref: 14960600 J Neurosci, 2004
